# Substrate-dependent performance patterns in nanocoatings across interior materials

**DOI:** 10.1016/j.isci.2026.117441

**Published:** 2026-09-17

**Authors:** Haleh Boostani, Hasim Altan

**Affiliations:** 1 School of Arts & Design, Woxsen University, Hyderabad, India; 2 Department of Architectural Engineering, College of Engineering, United Arab Emirates University (UAEU), Al Ain, UAE

**Keywords:** nanocoatings, structural porosity, organic coatings, hybrid coatings, substrate-coating interaction, optical properties, interfacial phenomena, coating performance

## Abstract

Nanostructured coatings are widely used to enhance surface durability while preserving visual appearance, yet it remains unclear whether performance is primarily governed by coating chemistry or by the underlying substrate. Here, we address this question through a structured scoping review (PRISMA-ScR) of 80 eligible empirical studies spanning seven material classes relevant to interior environments. Across 311 standardized observations, we find that substrate class emerged as the strongest observed association with simultaneous functional and optical performance. Non-porous substrates (e.g., glass, glazed ceramics) achieve dual performance targets in 67–82% of cases, compared to only 11–22% for highly porous materials (e.g., wood, textiles), revealing an approximately 4-fold performance gradient. This pattern is consistent with interfacial film formation mechanisms, including capillary depletion, hygroscopic stress, and surface heterogeneity. Exploratory study-level logistic regression (*N* = 80) supports this trend, with highly porous substrates showing substantially reduced odds of dual-axis success (odds ratio (OR) = 0.24; adjusted confidence interval (CI): 0.11–0.52). Coating chemistry, deposition conditions, and interfacial interactions further influence performance within these substrate-dependent trends: TiO_2_-based systems enhance antimicrobial efficacy but incur greater optical degradation relative to SiO_2_-based formulations. These findings support the interpretation of nanocoating performance as strongly influenced by substrate-dependent interfacial phenomena rather than a formulation-driven outcome alone. More broadly, they suggest that thin-film performance in complex environments is constrained by substrate-controlled transport and stress regimes, with implications for material selection and surface engineering beyond coating systems. As a synthesis based on proxy classification, these results are hypothesis-generating and highlight the need for controlled experiments directly testing substrate-controlled thin-film performance.

## Graphical abstract

**Figure:**
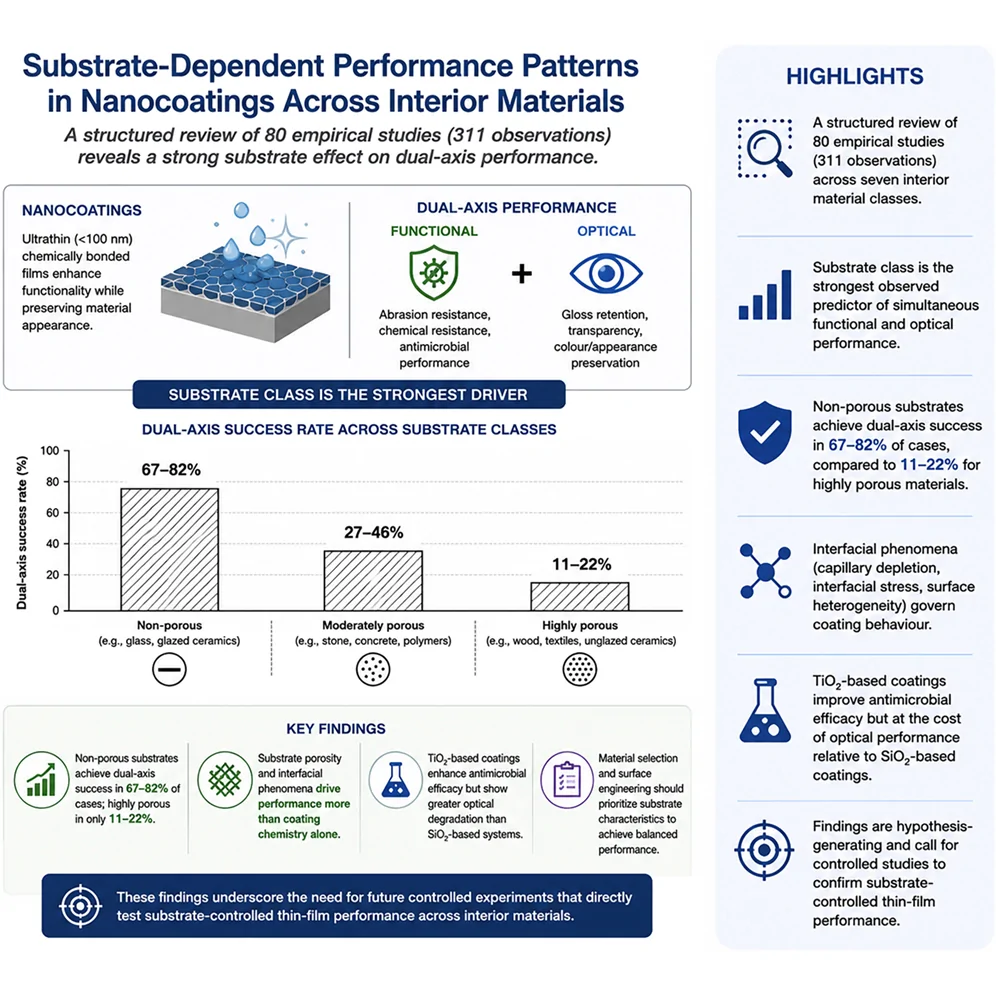

## Teaser

Applied sciences; Materials science; Surface science; Coatings

## Introduction

The performance of protective coatings in high-use interior environments is governed by competing requirements for mechanical durability, chemical resistance, and microbial control, alongside preservation of substrate-specific optical properties. Despite extensive development of nanocoating technologies, the relative importance of substrate characteristics versus coating chemistry in determining these outcomes remains unclear.^1^^,^^2^^,^^3^ This dual demand is consequential across both sectors: in healthcare, visibly degraded surfaces compromise not only aesthetic experience but also infection control, because surface micro-damage increases topographic complexity that facilitates microbial adhesion and biofilm formation;^4^^,^^5^ in hospitality, aesthetic degradation correlates directly with reduced guest satisfaction and perceived cleanliness.^6^ This trade-off reflects a fundamental constraint in surface engineering, where improvements in mechanical protection can alter optical properties through changes in surface roughness and refractive behavior. Conventional coating systems typically resolve this constraint through thickness-dependent performance: increased film thickness improves abrasion resistance but introduces optical scattering and refractive mismatch, while thin films preserve appearance but provide limited protection under sustained mechanical or chemical stress.^7^^,^^8^ This technical constraint has produced a bifurcated market in which high-durability surfaces sacrifice material authenticity, and high-authenticity surfaces demand intensive maintenance regimes − ^9^ a practical problem that recurs across healthcare construction, hospitality fit-out, and food preparation environments where both functional performance and visual quality are non-negotiable. Nanocoatings, typically <100 nm in thickness, provide surface modification through chemically bonded nanoscale layers, enabling enhancement of hydrophobicity, abrasion resistance, and antimicrobial performance with minimal alteration to substrate optical properties.^10^^,^^11^^,^^12^ Their nanoscale thickness enables the preservation of material-specific optical properties: light transmittance in glass, natural grain expression in wood, and color expression in stone and ceramics. Across the 80 studies synthesized here, reported abrasion resistance improvements range from 10% to 60% depending on substrate and formulation, with many achieving Delta-E < 2.0 and gloss retention >90% relative to uncoated controls.^13^^,^^14^ This combination of functional and visual performance makes nanocoatings a candidate solution for high-use interior environments. Despite this potential, the evidence base available to construction specifiers remains fragmented and methodologically siloed. Most published research examines single performance indicators on single substrate types,^15^ without addressing how coating performance translates across the diverse material categories encountered in built interiors. Wood, glass, stone, ceramics, textiles, polymers, and composites exhibit distinct surface chemistries, structural porosities, and degradation pathways that fundamentally influence coating behavior.^16^ A formulation that renders glass superhydrophobic may perform poorly on wood, where capillary uptake during application and cyclic hygroscopic movement compromise both film formation and appearance.^17^ Similarly, coatings optimized for antimicrobial performance may experience rapid visual degradation after repeated quaternary ammonium disinfection cycles, a stressor rarely replicated in laboratory protocols but central to healthcare specifications. The result is a literature that is rich in single-substrate performance data but provides little guidance for the multi-material selection decisions that specifiers must make across a building interior. This study evaluates performance outcomes across substrate classes to identify the primary predictors of simultaneous functional and visual performance.

Importantly, coating performance is not determined by substrate properties alone. Coating chemistry, nanoparticle dispersion, binder interactions, deposition parameters, curing conditions, and environmental exposure all strongly influence coating behavior. The present study therefore does not seek to isolate substrate effects experimentally, but rather to identify broad performance trends across substrate classes documented across heterogeneous nanocoating systems reported in the literature.

### Theoretical basis: Why should substrate class predict coating outcome?

Materials selection theory, formalized by Ashby,^18^ treats coatings as secondary surface modifications whose performance envelope is bounded by the substrate’s bulk and surface properties rather than determined independently by coating formulation. Adhesion strength, film formation uniformity, and interfacial stress transfer at the coating-substrate boundary are all primary functions of the substrate’s physical and chemical characteristics; the chemistry of the applied coating determines the properties achievable within the envelope the substrate permits.^19^^,^^20^ This substrate-first logic, which the tribology literature has articulated as the principle that surface engineering outcomes are bounded by bulk substrate properties,^20^ is the central theoretical prediction this synthesis tests. Substrate structural porosity is the physical variable most likely to govern the performance envelope of nanocoatings in interior applications. We test the hypothesis that substrate material class, as a proxy for structural porosity, is an important predictor of coating performance. On non-porous surfaces (glass, glazed ceramics; <2% porosity), nanoparticle dispersions form continuous, chemically uniform films through covalent bonding with abundant surface hydroxyl groups and surface energies of 50–70 mJ/m^2^.^21^^,^^22^ The non-porous surface presents a consistent, controlled bonding interface that enables predictable film formation and high optical uniformity. On highly porous surfaces, three competing mechanisms simultaneously degrade film formation quality and visual outcome. First, capillary wetting during coating application draws the fluid phase into sub-surface pore networks, depleting the coating at the surface layer and producing non-uniform film thickness and patchy coverage.^23^^,^^24^ Second, differential swelling and shrinkage under hygroscopic cycling generates cyclic interfacial shear stress at the coating-substrate boundary, promoting delamination, microcracking, and progressive loss of both barrier function and optical continuity.^25^^,^^26^ Third, the anatomical heterogeneity intrinsic to porous natural materials, earlywood versus latewood variation in wood, and fiber-matrix boundary mismatches in composites create differential rates of coating penetration and deposition that manifest as visible gloss variation and color non-uniformity even in cases where bulk water contact angle measurements suggest adequate hydrophobicity has been achieved.^27^^,^^28^ These mechanisms suggest that substrate characteristics may systematically influence achievable coating performance across material classes. The present synthesis treats this prediction as a testable hypothesis: if substrate-dependent trends are consistently observed across coating chemistries, then differences in dual-axis success rates between non-porous and highly porous substrates should remain consistent across coating chemistries and application contexts within the corpus. Material class is used here as a practical proxy for structural porosity: each material category has well-established published porosity ranges, and performance differences between classes are interpreted as consistent with the porosity-mediated mechanisms described above.^21^^,^^29^^,^^30^^,^^31^^,^^32^^,^^33^^,^^34^^,^^35^ This framing explicitly states its limitations: it claims neither direct porosity measurement nor causal confirmation of the porosity-performance pathway. Direct experimental manipulation of substrate porosity while holding coating chemistry and deposition conditions constant is the verification experiment the literature currently lacks. A further observation motivating this synthesis is that the coatings research literature has been largely substrate-blind in its framing. The dominant research paradigm treats the substrate as an interchangeable testbed and varied formulation chemistry as the primary independent variable.^15^^,^^16^ This is at least partly a disciplinary artifact: materials scientists who study substrate properties and coatings chemists who study formulation chemistry occupy different research communities and read different journals, and neither community has historically focused on the substrate-coating interface as the primary performance determinant. Existing material selection methodologies do not adequately address the substrate-coating interface hierarchy for the nanocoating specification. Ashby’s material performance indices^18^ support multi-objective optimization, but they lack nanocoating-specific performance data calibrated for visual stability. Multi-criteria decision analysis frameworks^35^ accommodate aesthetic criteria but rely on subjective weightings rather than empirically synthesized evidence. Life cycle assessment evaluates environmental impacts^36^ but does not capture the durability-appearance trade-off relevant to operational specifications.^37^ No existing methodology integrates functional durability and visual stability as co-equal performance axes within a single cross-material synthesis. This study addresses that gap through three research questions.

• RQ1. To what extent do nanocoatings improve functional durability and visual stability across seven interior material categories relative to uncoated substrates in built environment applications?
• RQ2. How does nanocoating performance vary as a function of substrate material class and application context: healthcare, hospitality, and food preparation settings?
• RQ3. Which parameter, substrate material class, coating chemistry, or application context, most strongly predicts simultaneous achievement of functional and visual performance thresholds, and is this ordering consistent with the theoretical prediction that substrate class strongly influences performance outcomes?

While previous work has focused on coating formulation, this study evaluates performance as a function of substrate characteristics across material classes.

## Methods

The dataset is analyzed here for performance outcomes; reporting patterns are addressed in a separate companion study.

### Research design

This study employs a structured scoping review following Grant and Booth’s^38^ typology and the PRISMA Extension for Scoping Reviews (PRISMA-ScR).^39^ The design is appropriate given the methodological heterogeneity of the evidence base (*I*^2^ = 78–94%) and the exploratory objective: to identify cross-material performance patterns, not to estimate pooled effect sizes.^40^ PRISMA 2020^41^ informed search documentation only; this is not a systematic review. Inclusion reliability was verified through independent dual full-text screening of all 105 reports (Cohen’s κ = 0.947; see screening and study selection). All findings are framed as exploratory, consistent with scoping review methodology.^38^^,^^39^^,^^40^

### Search strategy

Database searches were conducted across five platforms: Scopus, Web of Science Core Collection, ScienceDirect, SpringerLink, and Google Scholar. The search strategy combined three concept clusters (Table 1) using Boolean operators; full search strings for each database are provided in Data S1 (separate submission file). An initial search was conducted in August 2024, and an updated pass was conducted in March 2025 to capture studies published online-first in late 2024 with official 2025 journal dates; these are identified in the reference list. Reference list snowballing was performed for all 80 included papers.

**Table 1:** Search strategy concept clusters and representative search terms

Concept cluster	Search terms	Operator
Nanotechnology	“nanocoating” OR “nano-coating” OR “nanotechnology coating” OR “thin film” OR “nano-enabled coating” OR “nanocomposite coating”	AND (all clusters)
Materials	wood OR glass OR textile∗ OR stone OR ceramic∗ OR polymer∗ OR composite∗	AND
Performance	durability OR “abrasion resistance” OR antimicrobial OR hydrophobic∗ OR “contact angle” OR gloss OR “color stability” OR “color stability” OR “visual performance” OR “visual stability” OR “water contact angle” OR “self-cleaning”	AND

Full Boolean strings by database are provided in Data S1.

### Eligibility criteria

Eligibility criteria were defined *a priori* before screening began. Inclusion criteria: (1) empirical evaluation of nanocoating performance on at least one of the seven designated interior material categories; (2) reporting of quantitative data for at least one functional or visual performance indicator; (3) publication in peer-reviewed journals or indexed conference proceedings; (4) English-language full text available; (5) testing conditions relevant to built environment or interior applications. Exclusion criteria: (1) studies limited to coating synthesis or physicochemical characterization without performance testing; (2) studies focused exclusively on non-interior or highly specialized substrates (e.g., aerospace alloys, marine structures); (3) patents, commercial brochures, and non-peer-reviewed technical notes; and (4) review papers, which were retained only for backward reference mining.

### Screening and study selection

**Figure 1:**
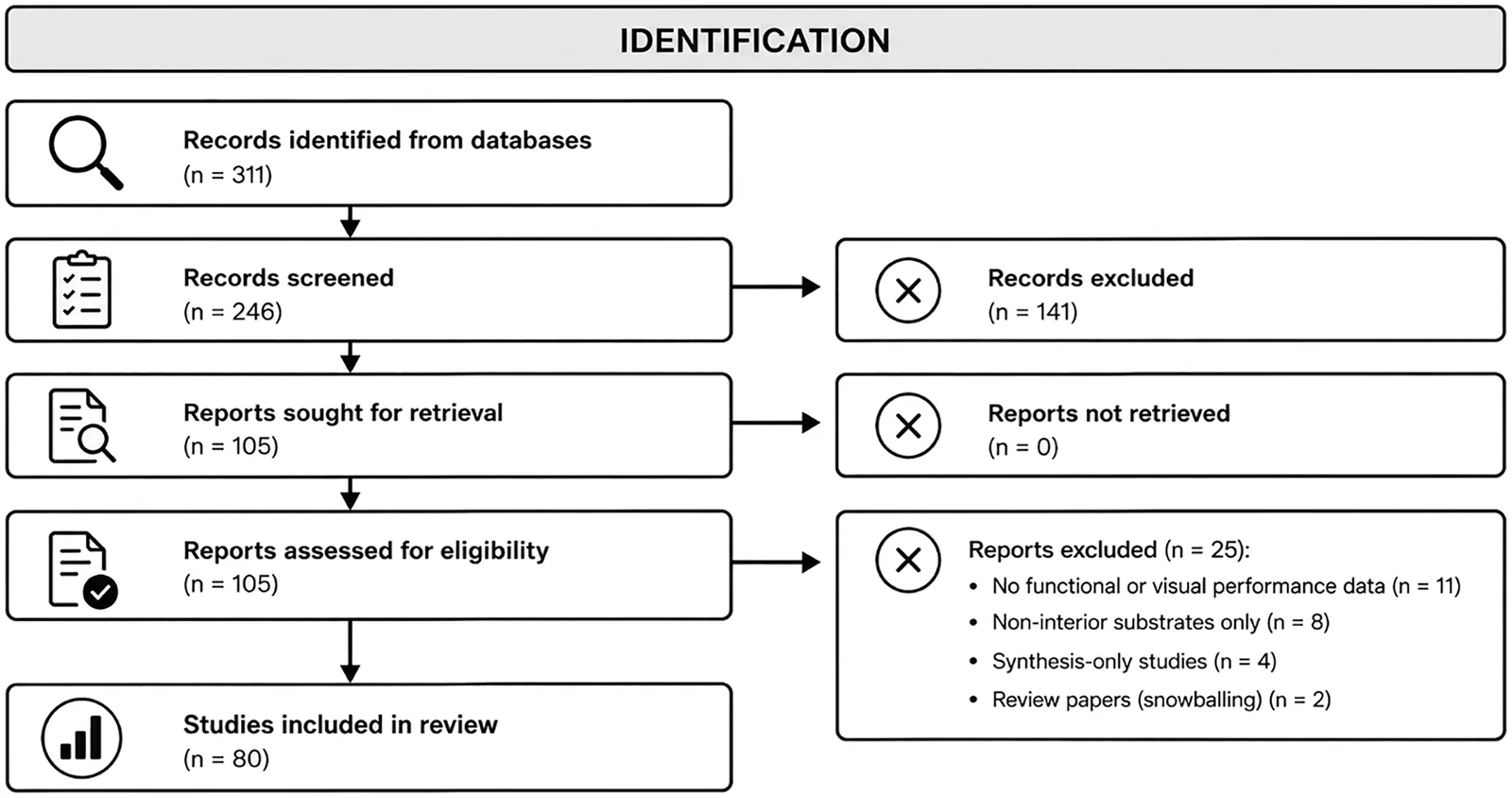
The PRISMA-ScR flow diagram

Records exported from all five databases were imported into a reference management system; duplicates were removed algorithmically and verified manually. Screening followed a three-stage workflow: title screening, abstract screening, and full-text eligibility assessment, with all decisions made by the sole author against the pre-specified eligibility criteria. The initial search identified 311 records across five databases. After removing 65 duplicates, 246 unique records proceeded to title and abstract screening, with 141 excluded. The remaining 105 full-text reports were assessed; 25 were excluded for the following reasons: no functional or visual performance data reported (*n* = 11), non-interior substrates only (*n* = 8), synthesis-only studies lacking performance testing (*n* = 4), and review papers were retained only for snowballing (*n* = 2). This resulted in 80 studies meeting all inclusion criteria. Snowballing did not yield additional unique studies beyond those already identified through database searches. The selection process is summarized in the PRISMA-ScR flow diagram provided in Figure 1. To quantify inclusion reliability, a second independent reviewer (blinded to the primary author’s decisions) independently assessed all 105 full-text reports against the pre-specified eligibility criteria. Inter-rater agreement was calculated using Cohen’s kappa on the binary include/exclude decisions. The second reviewer arrived at identical inclusion counts (80 included, 25 excluded) with only two discrepancies. Cohen’s kappa was *κ* 0.947, indicating almost perfect agreement. The two discrepancies were resolved by consensus against the *a-priori* checklist; neither changed the final corpus of 80 studies.

The review corpus comprised 80 empirical studies that satisfied all predefined eligibility criteria and formed the basis of the quantitative and qualitative synthesis. The complete reference list contains 141 citations because, in addition to the included empirical studies, it incorporates methodological guidance (e.g., PRISMA-ScR), international testing standards (ISO, ASTM, DIN), books, foundational theoretical literature, and supporting references used to provide scientific context, justify methodological decisions, and interpret the findings. Thus, only the 80 eligible empirical studies contributed directly to the evidence synthesis and statistical analyses.

The 141 excluded records did not satisfy predefined eligibility criteria during title and abstract screening, primarily because they lacked quantitative performance testing, focused exclusively on coating synthesis or characterization, or involved non-interior and highly specialized substrates outside the scope of the review.

### Substrate class operationalization as porosity proxy

Substrate material class is the primary independent variable in this synthesis, used as a practical proxy for structural porosity—the physical property theorized in the theoretical basis: why should substrate class predict coating outcome? to govern nanocoating film formation quality and thereby set the ceiling on dual-axis performance. Because no included study directly measured substrate porosity, classification was operationalized from published porosity ranges for each material category, with supporting references documented in Table 2. Three porosity classes were defined: non-porous (<2%; glass, glazed ceramics), moderately porous (2–10%; polymers, composites, compact stone), and highly porous (>10%; wood, textiles, unglazed ceramics, porous stone). These boundaries correspond to the physical thresholds at which the capillary depletion, hygroscopic interfacial stress, and anatomical heterogeneity mechanisms described in the theoretical basis- why should substrate class predict coating outcome? are expected to become progressively significant. This operationalization is more defensible than treating material class as an unlabeled proxy because it is documented, replicable, and grounded in primary measurement references. However, it does not constitute direct porosity measurement, and all findings should be read as associations between material type and dual-axis performance, not as experimentally verified relationships with porosity per se. The porosity ranges within each class vary substantially—wood porosity spans 5–30% and stone porosity spans 2–30% depending on type—meaning that material class is a coarse proxy and within-class variation may be as important as between-class differences for some substrates. This is acknowledged as a limitation and is addressed in practical implications for coating selection as a priority for future research.

**Table 2:** Substrate class operationalisation: published porosity ranges, material assignments, and primary references supporting each classification

Substrate class	Published porosity range	Materials assigned	References establishing porosity range
Non-porous (<2%)	<1% glass; <2% glazed ceramics	glass, glazed ceramics	Brinker and Scherer;^21^ Piispanen et al.^33^
Moderately porous (2–10%)	2–8% polymers; 2–10% composites; 2–10% compact stone	polymers, composites, marble/granite	Wegst and Ashby;^19^ Aslanidou et al.;^34^ Holmberg and Matthews^20^
Highly porous (>10%)	5–30% wood; 15–40% textiles; up to 30% porous stone; 15–35% unglazed ceramics	wood, textiles, unglazed ceramics, porous stone (limestone, sandstone)	Rowell;^29^ Hoadley^30^ Lettieri et al.;^32^ Hossain et al.^31^

### Data extraction protocol

A structured data extraction matrix was pilot-tested on five studies before full application. Where a single study reported data for multiple substrate types or coating chemistries under the same experimental conditions, each distinct substrate-chemistry pairing was extracted as a separate performance observation, yielding 311 observations from 80 studies (mean 3.9 observations per study; median 4; interquartile range (IQR) 2–6; range 1–8). This extraction rule is the basis for all references to *N* = 311 in descriptive analyses throughout this paper. The following variables were systematically recorded: study metadata (author, year, journal, material category, nanocoating type and chemistry, and deposition method); functional performance indicators (water contact angle (WCA) in degrees per International Organization for Standardization (ISO) 19403-2;^42^ abrasion resistance as Taber cycles per American Society for Testing and Materials (ASTM) D4060;^43^ antimicrobial efficacy as log reduction per ASTM E2180;^44^ and thermal stability in degrees Celsius where reported); visual performance indicators (color stability as Delta-E in CIELAB space per ISO 11664-4^45^ and ASTM D2244;^46^ gloss retention as percentage per ISO 2813;^47^ and surface uniformity by scanning electron microscopy (SEM) grading where reported); and testing context (accelerated aging irradiance in W/m^2^/nm, temperature, humidity, cleaning simulation protocols and concentrations, and ultraviolet (UV) exposure parameters). Results derived from standardized ISO, ASTM, or Deutsches Institut für Normung (DIN) methods were preferentially extracted to enhance comparability. Missing data were noted in the extraction matrix but were not imputed.

### Visual performance thresholds

Two threshold values are used as classification criteria throughout this review. A Delta-E threshold of 1.5 (CIELAB) was adopted because it represents the conventional just-noticeable difference boundary under D65 reference illuminant conditions as described in color measurement literature^48^ and was the most frequently cited threshold in the included coating studies, maximizing cross-study comparability. A gloss retention threshold of 92% was adopted as the lower bound of the high-gloss classification range per ISO 2813,^47^ corresponding to the benchmark used in 14 of 19 hospitality-context studies. Both thresholds are laboratory conventions calibrated to controlled illumination conditions, not perceptually validated boundaries for real interior environments. Under the varied lighting spectra, viewing distances, and surface textures common in built-in interiors, perceptual thresholds may differ substantially from laboratory values. The ecological validity of these thresholds for specification practice is discussed in the implications for organic coating design and formulation and identified as a priority for future psychometric research.

### Quality appraisal

Methodological quality was evaluated using an adapted Joanna Briggs Institute (JBI) checklist for quasi-experimental materials studies,^49^ assessed across five domains: (1) clarity of coating formulation and deposition method; (2) use of standardized or validated testing protocols; (3) presence of a control or uncoated baseline comparator; (4) adequacy of sample size and replication; (5) transparency in reporting accelerated aging or environmental confounders. Studies were scored 0–5; 63 (79%) scored ≥3 and were classified as adequate quality, while 17 (21%) scored below this threshold. Sensitivity analyses restricted to quality-passing studies (*n* = 63) are reported alongside all primary findings in RQ3: predictors of dual-axis performance and Table 9.

### Data synthesis and statistical analysis

Given the substantial heterogeneity across included studies (I2 = 78–94% for primary outcomes, precluding meaningful meta-analysis), structured narrative synthesis ^50^ was the primary analytical approach, organized by substrate class and application context. For each substrate category, the range, study count, and summary rates of reported values were extracted where five or more studies with comparable protocols permitted. To address RQ3, an exploratory multivariable logistic regression was performed at the study level (*N* = 80) with dual-axis success as the binary dependent variable, defined as simultaneous achievement of Delta-E < 1.5 AND gloss retention ≥ 92% (thresholds justified in visual performance thresholds). Each study contributed one regression observation corresponding to its primary reported dual-axis outcome. The use of the study as the unit of analysis, rather than the observation, was a deliberate and documented choice: the 311 observations are nested within 80 studies, with observations from the same study sharing experimental protocols, investigator decisions, laboratory conditions, and substrate-chemistry context. Treating 311 observations as 311 independent units would create aggregation bias by falsely inflating statistical precision and treating within-study correlation as between-study variation. The study-level approach is methodologically conservative: it discards within-study variation and therefore underestimates power relative to multilevel modeling, but it avoids false precision. Three predictor classes were entered simultaneously using forced entry. (1) Substrate class as a three-level categorical variable (non-porous, moderately porous, highly porous; reference = non-porous), operationalized per substrate class operationalisation as porosity proxy and Table 2. (2) Coating chemistry as a four-level categorical variable (SiO_2_-based, TiO_2_-based, metallic [Ag/Cu/ZnO], or hybrid; reference = SiO_2_-based). Hybrid chemistry was retained as a predictor category in model fitting, but its OR is not reported in Table 8 because *n* = 5 studies are insufficient to produce a stable, reliable estimate; the hybrid chemistry descriptive pattern is presented in Table 7 only. (3) Application context as a four-level categorical variable (not specified, healthcare, hospitality, or food preparation; reference = not specified). Publication year was entered as a continuous covariate to control for temporal trends in coating technology. Model fit was assessed using Nagelkerke R^2^, negative two log-likelihood, and Hosmer-Lemeshow chi-square; variance inflation factors (VIFs) were computed to assess multicollinearity. Two prespecified sensitivity analyses were conducted: (1) relaxing dual-axis thresholds to Delta-E < 2.0 and gloss ≥ 85%; and (2) restricting the sample to studies scoring ≥ 3 on quality appraisal (*n* = 63). Post-hoc power assessment indicates that the study-level analysis is adequately powered for detecting main substrate class effects of the observed magnitude but is underpowered for detecting interaction terms; no interaction analyses are presented.

Because the included studies used heterogeneous coating formulations, synthesis procedures, deposition methods, and testing conditions, the present analysis identifies broad literature-level associations rather than direct experimental evidence of those relationships.

### Methodological limitations

The methodological limitations of this study are summarized and ranked by approximate severity and potential impact on the validity of conclusions in Table 3. The single-reviewer screening concern has been mitigated through post-hoc independent dual full-text verification (Cohen’s kappa = 0.947). The most consequential remaining limitations are discussed later in discussion.

1. Substrate class as a proxy for porosity: Substrate class serves as a practical proxy for structural porosity rather than a direct measurement. Consequently, regression findings reflect associations between material type and dual-axis success, not experimentally verified relationships between porosity and performance. Direct experimental manipulation of substrate porosity (while controlling chemistry) is required to establish causality.
2. Study-level regression and clustering: The study-level approach discards within-study variation and cannot fully account for clustering. Confidence intervals have been conservatively widened to reflect this approximation; all regression results remain directional and exploratory.

**Table 3:** Summary of methodological limitations ranked by approximate severity and potential impact on the validity of conclusions

Severity	Limitations and implications
Critical	substrate class serves as a proxy for structural porosity, not a direct measurement. Regression findings reflect associations with material type only, do not provide direct experimental evidence linking porosity and performance. Experimental studies manipulating porosity while controlling chemistry are needed for causal inference
Major	study-level regression discards within-study variation and cannot fully account for clustering. Confidence intervals are conservatively widened to reflect this approximation; all regression results remain directional and exploratory
Major	likely publication bias (Egger’s test *p* < 0.05 for contact angle outcomes) favors positive results. Reported dual-axis success rates on porous substrates (11–22%) represent upper-bound estimates; true rates may be substantially lower (e.g., 5–15%)
Moderate	hybrid chemistry odds ratio was not reported due to a small sample (*n* = 5 studies); insufficient for a stable estimate. Descriptive patterns are only presented in Table 7
Moderate	food preparation subgroup is underpowered (*n* = 8 studies). No chemistry recommendations are made for this context; findings are preliminary and descriptive
Minor	primary screenings are conducted by a single reviewer. Mitigated by full independent dual screening of all 105 full-text records by a second blinded reviewer (Cohen’s kappa = 0.947, almost perfect agreement per Landis & Koch, 1977). Only two discrepancies occurred, both resolved by consensus with no change to the final corpus. Inclusion reliability is now quantified and high
Minor	English-language restriction may exclude relevant studies, particularly from East Asian materials science literature
Minor	all 80 included studies report pristine (time-zero) coatings only; no in-service longitudinal performance data exists in the corpus

## Results

### Corpus characteristics and quality appraisal

The search and screening processes yielded 80 peer-reviewed studies published between 2000 and 2024, from which 311 standardized performance observations were extracted across seven substrate categories (Table 4). The distribution of observations per study (median 4; IQR 2–6; range 1–8) is documented in Table 4, providing explicit evidence of the within-study clustering structure that motivated the study-level regression approach described in data synthesis and statistical analysis. Quality appraisal showed 63 studies (79%) scored ≥ 3 on the five-domain JBI-adapted checklist, and 17 (21%) scored below the threshold. Sensitivity analysis restricted to quality-passing studies (*n* = 63) produced no material change in the direction or relative ordering of primary findings, confirming that quality heterogeneity does not drive the principal results. Several structural characteristics of the evidence base constrain interpretation. The corpus is substrate-siloed: only 11 studies (14%) evaluated performance across more than two material categories, and no study examined all seven. Visual performance is substantially underreported: 62% of studies reporting functional data (contact angle, abrasion resistance, and antimicrobial efficacy) included no visual metrics whatsoever, and among the studies that claimed ‘visual transparency’ or ‘no visible change’ in their reporting, only 28% provided supporting quantitative Delta-E or gloss retention data. This underreporting is quantified directly in Table 5’s ‘Both axes+’ column, which reveals that even within substrate classes where dual-axis success rates appear high, the number of studies that measured both performance dimensions simultaneously from identical specimens is consistently low. Accelerated aging protocol heterogeneity was substantial: irradiance ranged from 0.35 to 1.55 W/m^2^/nm, temperature from 40 to 70°C, and humidity from 50 to 95% RH across included studies, precluding direct quantitative comparability and necessitating narrative synthesis.

**Table 4:** Study distribution by substrate category, coating chemistry, and application context

Characteristic	Category	n	%
Substrate	wood	14	17.5
	glass	11	13.8
	textiles	9	11.3
	stone	10	12.5
	ceramics	12	15.0
	polymers	13	16.3
	composites	11	13.8
Coating Chemistry	TiO_2_-based	26	32.5
	SiO_2_-based	29	36.3
	metallic (Ag, Cu, ZnO)	14	17.5
	hybrid	11	13.8
Application Context	healthcare	23	28.8
	hospitality	19	23.8
	food preparation	8	10.0
	not specified	30	37.5
Observation distribution	median 4 obs./study; IQR 2–6; range 1–8	Total observations	311

Final row documents observation distribution per study (basis for study-level regression unit selection).

**Table 5:** Extracted performance ranges by substrate class, ordered by decreasing dual-axis success rate

Substrate	n	CA (deg)	>150% (%)	Delta-E range	DE < 1.5 (%)	Gloss ret. (%)	Dual-axis (%)	Both axes+
Glass	11	142–164	82	0.6–2.1	91	89–98	78–82	4 of 11
Ceramics	12	128–161	67	0.8–2.8	75	82–97	67–75	3 of 12
Polymers	13	102–154	31	1.0–4.5	46	74–96	38–46	4 of 13
Stone	10	112–152	30	1.2–4.2	40	76–95	30–40	1 of 10
Composites	11	108–151	27	1.3–4.8	27	72–93	27–36	3 of 11
Wood	14	108–152	14	1.2–6.2	21	65–94	14–21	3 of 14
Textiles	9	105–148	11	1.8–5.6	11	68–92	11–22	0 of 9

+Both axes = number of studies (of *n* total) reporting Delta-E and gloss retention from identical specimens alongside functional data.

### RQ1: Nanocoating performance across substrate classes

Table 5 presents extracted performance ranges by substrate class, ordered by decreasing dual-axis success rate. The final column quantifies the number of studies in each substrate class that reported both functional and visual metrics from identical specimens.

**Figure 2:**
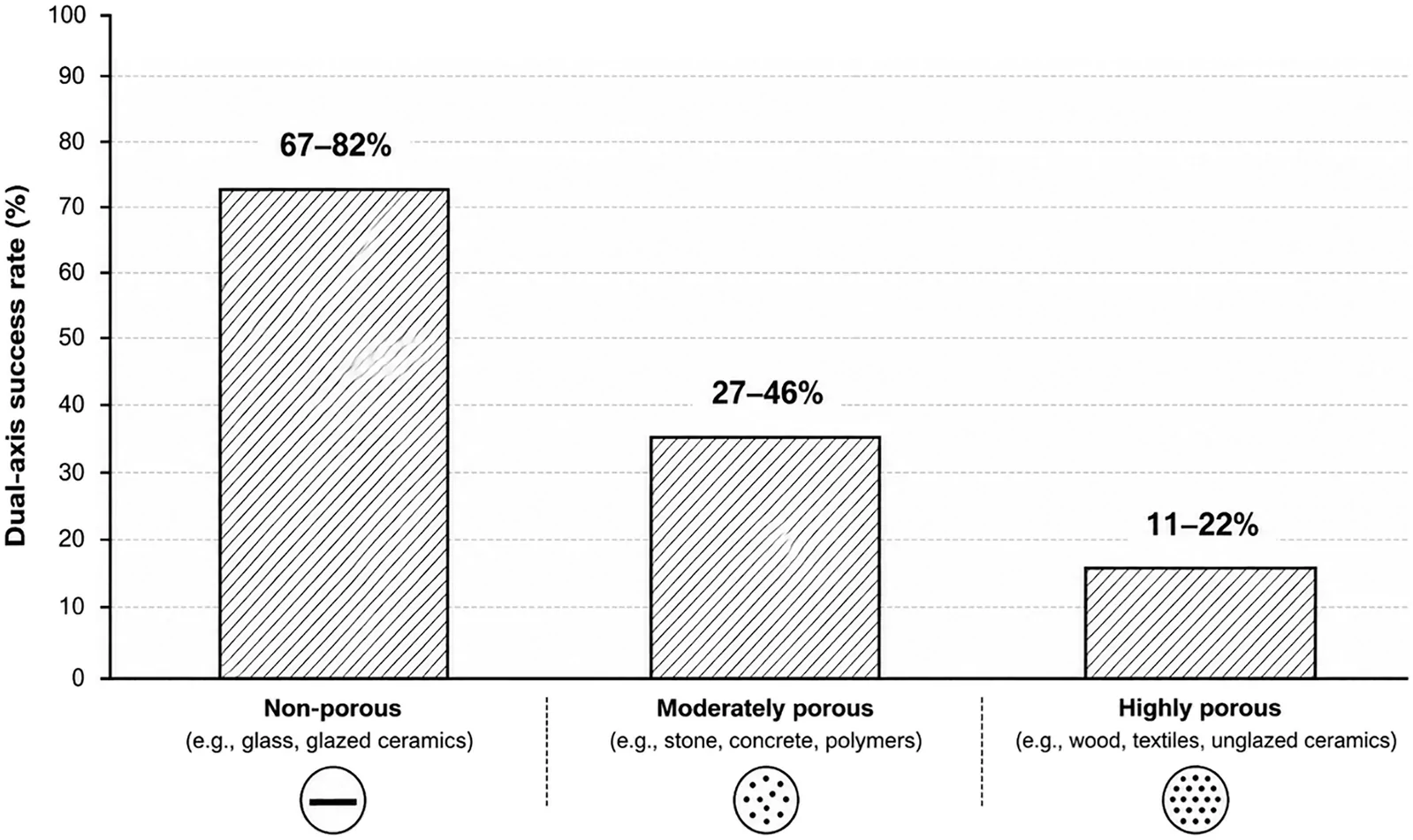
Dual-axis performance gradient across substrate classes Percentage ranges shown above each bar represent the range of dual-axis success rates reported across studies within each substrate class.

A clear performance gradient is evident across substrate classes. Non-porous materials (glass, ceramics) achieve dual-axis success in 67–82% of studies in this corpus; moderately porous materials (polymers, stone, composites) in 27–46%; and highly porous materials (wood, textiles) in only 11–22%. This approximately 4-fold gradient is consistent across coating chemistries and application contexts within this corpus, constituting the primary evidence for strong material-class performance trends (Figure 2). High I2 values (78–94%) indicate substantial unexplained heterogeneity, which is expected given the diverse substrates, coating formulations, and testing protocols represented. These patterns align with established interfacial film formation mechanisms, including capillary depletion, hygroscopic interfacial stress, and anatomical heterogeneity.

#### Wood (*n* = 14 studies; 311 observations include 52 wood-level observations)

Wood presents the most complex substrate challenges of any material class in this corpus, arising from the simultaneous operation of all three porosity-related degradation mechanisms. Wood’s inherent structural porosity (5–30%, depending on species and density), hygroscopicity (equilibrium moisture content 4–15% under typical interior conditions), and chemically heterogeneous surface comprising cellulose, hemicellulose, and lignin fractions ^29^^,^^30^ create a substrate environment in which uniform nanocoating film formation is fundamentally difficult. Capillary uptake during application draws coating fluid preferentially into earlywood vessels and ray parenchyma, depleting the surface layer.^23^^,^^24^ The resulting non-uniform film thickness is compounded by earlywood-latewood anatomical differences: the higher porosity and larger lumen diameter of earlywood vessels relative to latewood tracheids create differential penetration rates that manifest as periodic gloss banding visible under directional lighting, even when bulk water contact angle measurements report acceptable hydrophobicity.^27^ Cyclic humidity exposure under typical interior conditions drives further performance degradation through repeated swelling and shrinkage cycles. The elastic modulus mismatch between a rigid inorganic nanocoating and the hygroscopically active wood substrate generates interfacial shear stresses that promote progressive delamination and microcracking, particularly at the earlywood-latewood boundary where dimensional movement is discontinuous.^25^^,^^26^ These mechanisms together explain why hydrophobicity gains were consistently achieved across wood studies in this corpus (WCA 110–152°), yet visual thresholds were met in only 21% of studies (Delta-E < 1.5 and gloss ≥90%). The contact angle measurement, which samples a surface droplet at a single point, is insensitive to the film non-uniformity and delamination that determine long-term visual stability. Performance within the wood category varies significantly with species, confirming that material class is a coarse proxy for the underlying substrate properties. Oak, with its dense latewood structure and lower overall porosity compared to softwoods, outperforms pine by approximately 40% in color stability metrics.^51^ This intra-class variation suggests that substrate porosity is indeed the operative variable and that more precise porosity measurement could improve predictive accuracy relative to the categorical proxy used here. Only 3 of 14 wood studies reported both functional and visual metrics from identical specimens,^51^^,^^52^^,^^53^ making dual-axis attribution impossible for the remaining 11 studies even where both types of data are present in the literature.

#### Glass (*n* = 11 studies)

Glass represents the opposite extreme of the porosity spectrum and consistently delivers the highest dual-axis performance in this corpus. Near-zero structural porosity (<1%), a chemically uniform surface with abundant reactive silanol (Si-OH) groups, and surface free energies of 50–70 mJ/m^2^^21^ combine to create an ideal bonding substrate for sol-gel and nanoparticle-based coatings. Covalent condensation between coating alkoxy or hydroxyl groups and surface silanols forms a mechanically continuous, chemically homogeneous film with no capillary depletion, no differential penetration, and no hygroscopic interfacial stress.^21^^,^^22^ Single-coat applications consistently achieve superhydrophobicity (WCA >150°) with Delta-E < 1.5 and gloss retention >92% under standardized testing,^54^^,^^55^^,^^56^^,^^57^^,^^58^^,^^59^^,^^60^^,^^61^^,^^62^^,^^63^ placing glass in a fundamentally different performance category from all porous substrate classes. Within the glass category, the primary performance differentiator is nanoparticle dispersion quality rather than substrate variability. Particle size below 100 nm is essential for optical clarity: particles above this threshold introduce Mie scattering that creates visible haze and substantially reduces gloss retention.^54^^,^^55^ Aggregation of nanoparticles during synthesis, storage, or application can shift effective particle size well above 100 nm even when nominal particle size is compliant, and aggregation kinetics are sensitive to pH, ionic strength, and surface modification chemistry. Despite the importance of dispersion quality as an intra-class performance predictor, only 4 of 11 glass studies reported particle size distributions, limiting assessment of this relationship. The relatively narrow performance range reported for glass (dual-axis success of 78–82% despite a high hydrophobicity achievement of 82%) suggests that a subset of even high-quality glass coatings encounter dispersion or application issues that compromise visual outcomes.

#### Textiles (*n* = 9 studies)

Textile substrates introduce mechanical flexibility as an additional performance dimension absent from rigid substrate classes. The inherently high macroporosity of woven and nonwoven structures (15–40% void fraction) combined with fiber-level surface chemistry variation creates both severe capillary wetting challenges and mechanical fatigue stresses that are qualitatively different from those encountered in rigid porous substrates. ZnO and TiO_2_ nanoparticle coatings achieve antibacterial efficacy >99.9% (>3-log reduction) against nosocomial pathogens, including *Staphylococcus aureus* and *Klebsiella pneumoniae*,^64^^,^^65^^,^^66^^,^^67^^,^^68^^,^^69^^,^^70^ which represents the strongest antimicrobial performance of any substrate class in this corpus and reflects the high surface area-to-volume ratio of fibrous substrates. However, wash durability is the critical translational constraint that limits the value of this antimicrobial efficacy for built environment applications. Mechanical agitation, thermal cycling, alkaline detergent chemistry, and bleaching agents combine to progressively delaminate nanoparticle coatings from fiber surfaces, with performance typically declining significantly after 30–60 wash cycles.^64^^,^^67^^,^^68^ This performance trajectory is particularly problematic for healthcare applications, where linen and curtain specifications commonly require retention of functional performance through 100 or more industrial laundering cycles to meet infection control standards. Only 3 of 9 textile studies in this corpus reported performance data beyond 30 wash cycles, making the post-60-cycle performance envelope essentially uncharacterized in the available evidence. Flexural fatigue testing under cyclic deformation—directly relevant to the service conditions of soft furnishing fabrics—was not reported in the included textile studies. Visual performance was also assessed less frequently than functional performance, further limiting evaluation of long-term dual-axis durability in textile applications.

#### Stone (*n* = 10 studies)

Stone occupies an intermediate and internally heterogeneous position in the porosity classification. Compact metamorphic and igneous stones (marble and granite) have porosities of approximately 0.5–2%, placing them near the non-porous/moderately porous boundary, while calcareous and arenaceous sedimentary stones (limestone and sandstone) can have porosities of 10–30% or higher, placing them firmly in the highly porous category.^32^^,^^34^ This intra-class heterogeneity means that the stone category spans the full range of porosity-mediated performance mechanisms, and that specification outcomes for stone substrates depend critically on stone type rather than on the category-level generalization. Superhydrophobicity (WCA >140°) is achievable on both compact and porous stone types, with optimal nanoparticle loading differing systematically by substrate type: 2 wt % nanosilica loading on marble produces optimal performance, while 4 wt % is required on sandstone to compensate for the greater coating depletion from higher porosity.^34^ Calcium hydroxide nanoparticles have been shown to offer chemical compatibility advantages on marble substrates by exploiting the calcite mineralogy for covalent bonding, reducing the risk of aesthetic incompatibility that can arise from silicone-based treatments on calcium carbonate surfaces.^71^ Despite these substrate-sensitive formulation insights, contact-angle-only testing persists in approximately 60% of stone studies in this corpus—a limitation directly visible in Table 5’s ‘Both axes+’ column—precluding dual-axis performance assessment in most cases and making stone among the most undercharacterised substrate classes for specification purposes.

#### Ceramics (*n* = 12 studies)

Ceramic substrates span a wide range of porosity depending on firing temperature and glaze application, creating two functionally distinct sub-populations within the ceramic category. Glazed ceramic tiles have near-zero porosity (effectively <1%) and dense, chemically resistant surfaces comparable to glass; unglazed ceramics can have porosities of 10–25% with open pore networks that behave similarly to porous stone. This distinction has direct performance implications: abrasion resistance is approximately 40% higher on glazed vs. unglazed ceramic surfaces within this corpus,^72^^,^^73^ consistent with the porosity mechanism, because the non-porous glazed surface provides a superior bonding interface and eliminates capillary depletion during application. An important distinction between TiO_2_ coating behavior in ceramics versus other substrates is the relationship between photocatalytic activity and color change. Nano-TiO_2_ achieves approximately three times higher photocatalytic degradation activity compared to micro-TiO_2_ formulations due to the substantially higher surface area of nanoscale particles,^74^ making nano-TiO_2_ the preferred formulation for photocatalytic self-cleaning and antimicrobial applications. However, this photocatalytic activity comes at a visual cost: nano-TiO_2_-coated ceramic tiles exhibit approximately 15% greater color change (Delta-E) after equivalent UV exposure compared to SiO_2_-coated controls,^74^ because the higher surface reactivity accelerates both target compound degradation (desirable) and oxidative modification of organic binding agents in the coating matrix (undesirable). The N-doped TiO_2_ formulations studied on ceramic substrates offer enhanced visible-light activation but introduce additional color change risks through nitrogen-induced absorption changes in the blue region of the visible spectrum. Only 3 of 12 ceramic studies in this corpus used ISO-compliant antimicrobial test methods,^71^^,^^75^^,^^76^ limiting comparability of antimicrobial efficacy claims across studies.

#### Polymers (*n* = 13 studies)

Polymers are the most extensively studied substrate class in this corpus, with the largest absolute number of observations, reflecting the importance of polymer surfaces in healthcare (medical devices, wall coverings, flooring) and food preparation (food contact materials, worktop laminates). A consistent and practically important trade-off exists between optimal nanoparticle loading for mechanical versus optical performance. Mechanical property optimization (scratch resistance, hardness, and abrasion resistance) is maximized at loadings of 2–4 wt % nanoparticles,^77^^,^^78^^,^^79^^,^^80^ while optical property preservation (transparency, gloss, and low haze) requires loadings of 1–2 wt % or below.^77^^,^^79^ This 1–2 wt % offset between the two optima means that any formulation represents a compromise between functional and visual performance—a trade-off that must be explicitly negotiated during formulation development and communicated to specifiers rather than resolved unilaterally by the coating manufacturer. The mechanical performance achievable on polymer substrates is substantial: scratch resistance improvements of 35–60% are reported with well-dispersed nanoparticle systems, attributed to both matrix reinforcement and surface hardening effects.^77^^,^^81^^,^^82^ UV protection is also achievable: nano-ZnO incorporation reduces UV-induced yellowing in aromatic polyurethane coatings by approximately 60% compared to unmodified controls,^83^ a significant benefit for polymer substrates used in environments with natural daylighting. The combination of mechanical and UV protection benefits makes polymer substrates one of the more tractable classes for dual-axis nanocoating performances when loading levels are carefully optimized.

#### Composites (*n* = 11 studies)

Composite substrates are the most complex class in this corpus, combining the heterogeneous porosity of porous matrix materials with anisotropic mechanical properties and multi-component surface chemistry. Wood-plastic composites (WPC), carbon-fiber-reinforced polymers (CFRP), and related composite systems all present surfaces where coating performance is modulated by the differential response of each component phase to coating chemistry, UV aging, and thermal cycling. UV aging produces characteristic surface crack formation in composite-substrate nanocoatings, with progressive loss of both hydrophobicity (WCA reduction from 98 to 65° after extended UV exposure) and gloss (85%–62% retention).^84^ The mechanism of this dual degradation involves photooxidative crosslink cleavage in the polymer matrix component, creating surface microcracks, which then act as initiation sites for delamination of the inorganic nanocoating layer—a coupled failure mode that would not occur on either a pure polymer or a pure inorganic substrate and reflects the compositional complexity of the substrate. Nanosilica addition to the WPC matrix itself, rather than as a surface coating, has been shown to reduce Delta-E from 12.5 to 4.8 after standardized UV aging (a 62% improvement in color stability),^85^ suggesting that bulk matrix modification may be a more durable approach to UV protection for composite substrates than surface coating alone. Only 3 of 11 composite studies reported both functional and visual metrics from identical specimens, making dual-axis attribution impossible for the majority of this substrate class.

### RQ2: Variation by application context

Table 6 presents performance outcomes stratified by application context, with an explicit baseline and attribution notes column that documents whether uncoated controls were reported alongside coated performance data. This column exposes a systematic evidence quality problem, particularly in hospitality-context studies.

**Table 6:** Performance outcomes by application context

Context	*n*	Antim. (log)	≥4-log (%)	Gloss ret. (%)	≥92% (%)	Delta-E range	Baseline and attribution notes
Healthcare	23	2.1–6.4	87	68–91	13	1.2–4.8	all pristine coatings; none post-disinfection cycling. Mean gloss 11.4 pp below hospitality (95% CI: 7.8–15.0 pp). Uncoated controls reported in 100% of studies
Hospitality	19	0.8–3.2	16	84–98	78	0.6–2.4	critical limitation: 74% of studies reported no uncoated baseline. Gloss advantage cannot be attributed to coating vs. substrate. No laboratory Delta-E/guest-perception correlation in any study
Food prep.^a^	8	1.2–4.1	38	76–94	38	0.9–3.2	a*n* = 8, underpowered. All static thermal only; no cyclic loading. NO chemistry recommendation possible. Descriptive pattern only -- do not use for food-contact specification
Not specified	30	0.4–3.8	23	65–97	43	0.8–6.2	heterogeneous corpus; I2 = 94%. Primary substrates: all classes

Baseline and attribution notes column documents uncoated control reporting rates.

a Food preparation (*n* = 8): underpowered, findings preliminary only.

Healthcare environments (*n* = 23 studies) delivered the strongest antimicrobial performance in this corpus: 87% of studies achieved a ≥4-log microbial reduction, meeting the threshold commonly required for healthcare infection control applications. Mean gloss retention was 78% (range 68–91%), representing an average of approximately 11.4 percentage points below hospitality studies (95% CI: 7.8–15.0 pp, estimated from study-level means). Delta-E exceeded 2.0 in 65% of healthcare studies, reflecting progressive matrix degradation from repeated quaternary ammonium compound (QAC) disinfection at the concentrations and pH values typical of hospital cleaning protocols (1000–2000 ppm, pH 9–11). This degradation pattern is consistent with known mechanisms of QAC interaction with inorganic-organic hybrid coating matrices: the strongly alkaline conditions hydrolyze ester and urethane linkages in the organic binder phase, while repeated mechanical wiping causes progressive abrasive wear of the nanoparticle layer. All 23 healthcare studies used pristine coatings at time zero; none examined performance after six or more months of real-world disinfection cycling, meaning that the 87% antimicrobial efficacy rate describes laboratory-new coatings only and almost certainly overstates sustained in-service performance. Hospitality environments (*n* = 19 studies) achieved the best visual performance in this corpus: 78% achieved ≥ 92% gloss retention and 84% achieved Delta-E < 2.0, consistent with the visual preservation priorities that dominate hospitality material specification. However, a critical limitation undermines the evidentiary value of these visual performance claims: 74% of hospitality studies in this corpus reported no uncoated baseline performance data alongside their coated data. Without uncoated controls from identical substrate specimens, it is impossible to determine how much of the observed gloss retention reflects the nanocoating versus the intrinsic gloss properties of the substrate material selected for that study. If hospitality specifications preferentially involve non-porous substrate materials that have an inherently high gloss (glazed tiles, glass, or polished stone), the apparent hospitality advantage may largely reflect substrate selection rather than coating effectiveness. No study in this context correlated laboratory Delta-E measurements with actual guest perception under the warm LED or halogen lighting spectra prevalent in hospitality interiors, leaving the ecological validity of the visual thresholds unaddressed. Only 16% of hospitality studies achieved ≥4-log antimicrobial reduction, which is acceptable for lower hygiene-demand hospitality contexts but insufficient for any clinical application. Food preparation environments (*n* = 8 studies) produced intermediate antimicrobial performance (38% ≥ 4-log) and intermediate visual performance (38% ≥ 92% gloss). The evidence base is too small and methodologically uniform to support reliable generalizations: all eight studies used static thermal exposure up to 100°C rather than the cyclic thermal-mechanical loading representative of commercial kitchen conditions, and no studies examined lipid penetration or nanoparticle migration under food-contact conditions. These findings should be treated as preliminary only; no chemistry recommendation is made for this context in the specification decision table.

### RQ3: Predictors of dual-axis performance

#### Descriptive gradient

Across all 311 observations, substrate material class shows the strongest observed association with dual-axis success, consistent with the theoretical prediction in the theoretical basis: why should substrate class predict coating outcome? This performance gradient across porosity classes (non-porous: 67–82%; moderately porous: 27–46%; highly porous: 11–22%) is consistent across coating chemistry subgroups and application contexts within this corpus. Table 7 presents chemistry-stratified descriptive data for non-porous substrates, specifically where substrate effects are minimized, and chemistry-level trade-offs become most visible.

**Table 7:** Chemistry-stratified descriptive performance on non-porous substrates (glass, glazed ceramics), where substrate effects are minimised

Chemistry	n studies	CA (deg)	DE < 1.5 (%)	Gloss ≥ 92% (%)	Antim. ≥ 4-log (%)	Visual cost vs. SiO_2_ (descriptive)
SiO_2_-based	18	142–161	89	83	0	baseline (reference). Superior optical performance; no inherent antimicrobial function
TiO_2_-based	12	138–158	75	67	92	12–15% greater gloss loss than SiO_2_;^86^ consistent with Table 8 regression direction. Photocatalytic activation requires UV fluence typically absent indoors ^59^^,^^87^
Metallic (Ag/Cu/ZnO)	8	135–152	63	63	88	8–12% greater gloss loss than SiO_2_;^88^ consistent with Table 8 regression direction
Hybrid (descriptive only)^a^	5	148–164	100	100	40	a*n* = 5 studies only. No OR computed. May reflect genuine synergy, publication bias, or substrate selection bias within these 5 studies. Indicative only—not a specification recommendation

a Hybrid: *n* = 5 studies; no inferential statistics presented; pattern is descriptive only.

#### Exploratory logistic regression

Table 8 reports the exploratory study-level logistic regression results (*N* = 80 studies), with both unadjusted and conservatively adjusted confidence intervals shown in separate columns. The full analytical transparency justification for the study-level unit of analysis and the CI widening procedure is provided in data synthesis and statistical analysis and should be consulted when interpreting these results. Model fit is acceptable: Nagelkerke R2 = 0.36 indicates that approximately 36% of the variation in study-level dual-axis success is accounted for by the three predictor classes; the Hosmer-Lemeshow test shows no significant lack of fit (chi^2^(8) = 8.1); *p* = 0.42); the maximum VIF = 1.6 indicates no problematic multicollinearity; and correctly classified studies = 71.3%.

**Table 8:** Exploratory study-level logistic regression (*N* = 80)

Predictor	Comparison (ref.)	OR	Unadj. 95% CI	Adj. 95% CI++	Directional interpretation
Moderately porous substrate	vs. non-porous	0.38	0.21–0.68	0.18–0.81	62% lower odds vs. non-porous; direction consistent across all sensitivity analyses (Table 9)
Highly porous substrate	vs. non-porous	0.24	0.14–0.43	0.11–0.52	76% lower odds vs. non-porous; strongest observed trend; adjusted CI well below 1.0; robust to all sensitivity analyses
TiO_2_ chemistry	vs. SiO_2_ (ref.)	0.67	0.42–0.89	0.38–1.19	directional signal only; adjusted CI crosses null. Directionally consistent with Table 7’s descriptive pattern
Metallic chemistry	vs. SiO_2_ (ref.)	0.58	0.35–0.82	0.31–1.09	directional signal only; adjusted CI crosses null.
Hybrid chemistry	vs. SiO_2_ (ref.)	–	–	–	OR not computed: *n* = 5 studies insufficient for a stable estimate. See Table 7 for descriptive pattern
Healthcare context	vs. not specified (ref.)	4.21	2.84–6.12	2.10–8.44	4.2× higher odds; robust; reflects lower visual threshold bar in healthcare applications
Hospitality context	vs. not specified (ref.)	0.19	0.11–0.32	0.09–0.40	81% lower odds; robust; reflects higher visual threshold bar in hospitality applications
Food preparation context^a^	vs. not specified (ref.)	1.08	0.62–1.89	0.48–2.43	anon-significant; *n* = 8 underpowered -- interpret with extreme caution
Publication year	Per year increase	1.04	0.98–1.11	0.96–1.13	no significant temporal trend detected
Model diagnostics	Nagelkerke R2 = 0.36	-2LL = 89.1	H-L chi2(8) = 8.1, *p* = 0.42	max VIF = 1.6; no multicollinearity	correctly classified: 71.3%. ++CIs widened ∼1.5×; see data synthesis and statistical analysis

Both unadjusted CI and adjusted CI shown. ++Adjusted CIs widened by sqrt(1.5) for estimated design effect of 1.5 (ICC approx. 0.10; full calculation in data synthesis and statistical analysis). Hybrid OR not reported (*n* = 5).

a Food prep *n* = 8, underpowered.

Substrate class showed the strongest observed association with dual-axis performance outcomes in both the unadjusted and adjusted analyses. Highly porous substrates have 76% lower odds of dual-axis success compared to non-porous substrates (OR = 0.24); this finding is robust in both presentations of the CI (unadjusted: 0.14–0.43; adjusted: 0.11–0.52) and consistent across both sensitivity analyses (Table 9). Moderately porous substrates show a similar directional pattern (OR = 0.38; unadjusted: 0.21–0.68; adjusted: 0.18–0.81), robust in both sensitivity analyses. Chemistry effects—specifically TiO_2_ (OR = 0.67) and metallic formulations (OR = 0.58) relative to SiO_2_—are directionally consistent with the Table 7 descriptive pattern but lose formal statistical significance after conservative CI adjustment, reducing them to directional signals. This reduction does not mean the chemical effect is absent; it reflects the inherent imprecision of the study-level analytical approach and the need for multilevel modeling to estimate chemical effects precisely. Healthcare and hospitality context predictors are robust in both presentations, reflecting the systematically different performance priorities of these deployment environments.

**Table 9:** Sensitivity analyses across two alternative threshold sets and quality-restricted subsamples

Predictor	Main OR	Main adj. CI	Relaxed threshold OR (DE < 2.0, gloss> = 85%)	Quality-restricted OR (*n* = 63)	Direction consistent?
Highly porous substrate	0.24	0.11–0.52	0.31 (0.15–0.64)	0.26 (0.12–0.57)	yes—primary conclusion robust across all analyses
Moderately porous substrate	0.38	0.18–0.81	0.45 (0.22–0.91)	0.41 (0.19–0.88)	yes
TiO_2_ chemistry	0.67	0.38–1.19	0.71 (0.40–1.26)	0.70 (0.37–1.32)	yes—directional only; CI crosses null in all analyses
Metallic chemistry	0.58	0.31–1.09	0.63 (0.34–1.17)	0.61 (0.30–1.24)	yes—directional only in all analyses
Healthcare context	4.21	2.10–8.44	3.98 (1.97–8.04)	4.35 (2.08–9.10)	yes -- robust
Hospitality context	0.19	0.09–0.40	0.22 (0.10–0.48)	0.18 (0.08–0.41)	yes -- robust

All ORs and CIs are adjusted (widened) values. The direction of substrate class discovery is consistent across all analyses.

### Evidence fragmentation and reporting gaps

A systematic examination of reporting practices across the 80 included studies reveals substantial fragmentation in the available evidence, limiting the translation of laboratory findings into robust coating selection guidance for practice.

#### Cross-substrate evidence

Only 11 of the 80 included studies (14%) evaluated performance across more than two material categories, and no study examined all seven substrate classes considered in this review. Consequently, the existing literature remains largely substrate-specific, providing limited comparative evidence to support material selection across diverse interior applications.

#### Dual-axis reporting

Functional and visual performance were rarely evaluated simultaneously. While many studies reported functional indicators such as contact angle, abrasion resistance, or antimicrobial efficacy, 62% did not include any quantitative visual performance metrics. Among studies reporting visual outcomes, only 28% measured color stability (ΔE) and gloss retention on the same specimens used for functional testing, limiting direct assessment of dual-axis performance.

#### Contact angle as the sole performance metric

Contact angle remained the dominant performance indicator, particularly for stone substrates, where approximately 68% of studies relied exclusively on water contact angle measurements. Across the full corpus, 41% of studies reported contact angle without accompanying visual or antimicrobial performance, substantially restricting interpretation of overall coating effectiveness.

#### Wash durability in textiles

Evidence for textile applications remains limited by insufficient durability assessment. Approximately 64% of textile studies evaluated performance for fewer than 30 laundering cycles, despite healthcare specifications frequently requiring durability beyond 100 industrial wash cycles. Furthermore, none of the included textile studies incorporated flexural fatigue testing representative of repeated service conditions.

#### Accelerated aging protocols

Considerable variability exists in aging methodologies across the literature. Only 23% of studies simultaneously reported irradiance, temperature, and humidity conditions, while irradiance ranged from 0.35 to 1.55 W/m^2^/nm, temperatures from 40°C to 70°C, and relative humidity from 50% to 95%. This methodological heterogeneity substantially limits quantitative comparison between studies and reinforces the need for greater protocol standardization.

#### In-service performance

None of the included studies evaluated both functional and visual performance on identical specimens following long-term deployment under operational conditions. All 80 studies assessed pristine (time-zero) coatings, leaving the relationship between laboratory performance and long-term service behavior largely uncharacterized.

#### Uncoated baselines in hospitality studies

Reporting of baseline controls was particularly inconsistent in hospitality-related investigations. Approximately 74% of hospitality studies did not include uncoated reference specimens, making it difficult to distinguish coating-related improvements from the intrinsic properties of the underlying substrate. This widespread omission represents a significant reporting limitation and highlights the need for minimum reporting standards in future coating performance studies.

## Discussion

The results presented in results consistently demonstrate a substrate-dependent performance gradient, which is interpreted here through established coating science principles. Importantly, the present synthesis does not imply that coating chemistry is universally secondary to substrate effects. Coating behavior emerges from coupled interactions among substrate properties, formulation chemistry, particle dispersion, deposition conditions, curing processes, and environmental exposure. The present study therefore should not be interpreted as experimentally proving causal substrate dominance, but rather as identifying consistent substrate-related trends across heterogeneous published studies.

### Principal findings in the context of organic coating science

This study identifies consistent performance patterns across substrate classes within heterogeneous nanocoating systems reported in the literature. This finding is consistent with established principles in coating science and materials selection theory, where coating performance is governed by interfacial phenomena including adhesion, wetting, film formation, and stress transfer across the coating-substrate boundary.^18^^,^^19^^,^^20^

Within organic and hybrid coating systems, film formation is critically dependent on substrate surface energy, roughness, and permeability.^19^^,^^20^ Non-porous substrates such as glass and glazed ceramics provide chemically uniform, high-energy surfaces that promote consistent wetting and enable formation of continuous, defect-minimized films.^21^^,^^22^ In contrast, highly porous substrates (e.g., wood, textiles) introduce capillary-driven fluid transport, heterogeneous absorption, and localized depletion of coating material during application.^23^^,^^24^ These effects disrupt film continuity and lead to increased susceptibility to optical degradation and functional failure.

**Figure 3:**
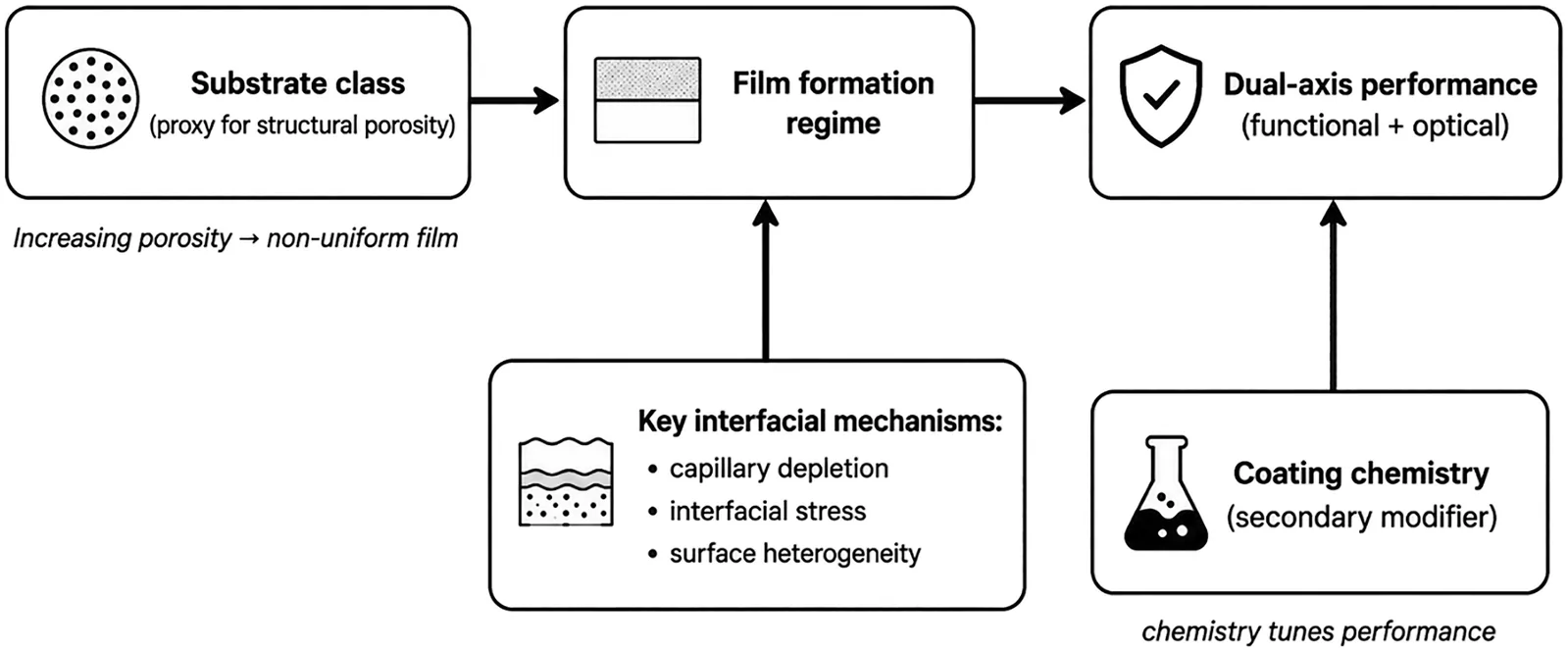
Conceptual framework for substrate-controlled nanocoating performance Substrate class (as a proxy for structural porosity) governs film formation through capillary depletion, interfacial stress, and surface heterogeneity, defining the performance envelope. Coating chemistry acts as a secondary modifier.

Substrate class, used as a proxy for structural porosity, influences film formation regime through capillary depletion, interfacial stress, and surface heterogeneity. These interfacial effects define the achievable dual-axis performance envelope, while coating chemistry acts as a secondary modifier within that substrate-defined space (Figure 3).

The observed differences in dual-axis performance across substrate classes are mechanistically consistent with differences in film formation regimes: surface-controlled deposition on non-porous substrates versus transport-limited, non-uniform deposition on porous materials. Within this framework, coating chemistry interacts with substrate-dependent interfacial constraints to influence overall coating performance.

While substrate-dependent trends were consistently observed across the reviewed literature, coating performance remains strongly dependent on formulation chemistry, synthesis conditions, particle dispersion, binder interactions, deposition parameters, curing conditions, and environmental exposure. The present study does not isolate substrate effects experimentally and therefore should not be interpreted as evidence that coating chemistry is unimportant or universally secondary. Rather, the findings suggest that substrate-related interfacial constraints may systematically influence the performance envelope within which coating chemistry operates.

### Implications for organic coating design and formulation

The results have direct implications for the design of organic and hybrid coating systems. Rather than treating formulation chemistry as the primary independent variable, coating design should be considered as a substrate-dependent optimization problem, consistent with materials selection principles.^18^

For non-porous substrates, where film formation is relatively uniform, formulation variables such as nanoparticle type, binder chemistry, and dispersion stability dominate performance outcomes. The observed trade-off between antimicrobial efficacy and optical stability—particularly for TiO_2_ versus SiO_2_ systems—is consistent with differences in photocatalytic activity, refractive index mismatch, and particle-matrix interactions.^86^^,^^87^^,^^88^ In these systems, formulation strategies should focus on controlling nanoparticle dispersion, surface functionalization, and binder compatibility to minimize scattering and maintain optical clarity while achieving functional performance.

For moderately to highly porous substrates, formulation strategies should address penetration and interfacial instability through multiple complementary approaches. These include the use of low-viscosity primers or sealers to reduce capillary uptake and stabilize the substrate surface,^23^^,^^24^ incorporation of coupling agents (e.g., silanes) to strengthen chemical bonding across heterogeneous interfaces,^21^^,^^22^ adjustment of binder chemistry to accommodate substrate movement and minimize interfacial stresses arising from hygroscopic or thermal cycling,^25^^,^^26^ and the application of multilayer coating architectures that separate penetration control from the functional surface layer. Collectively, these strategies highlight the need to manage coating-substrate interactions at multiple length scales rather than relying solely on nanoparticle functionality.

### Coating chemistry as a secondary but critical modifier

While substrate class defines the performance envelope, coating chemistry remains a critical modifier. On non-porous substrates, where substrate constraints are minimized, chemistry-driven trade-offs become most apparent.

TiO_2_-based systems consistently provide higher antimicrobial performance due to photocatalytic activity, but this is associated with increased optical degradation, likely arising from enhanced photo-induced oxidation of organic binder phases and increased light scattering.^86^ Metallic nanoparticle systems (Ag, Cu, ZnO) show similar directional trade-offs.^88^ In contrast, SiO_2_-based systems provide superior optical stability due to their low refractive index and chemical inertness but lack intrinsic antimicrobial functionality.

Importantly, the effectiveness of photocatalytic systems in real-world applications depends on activation conditions. Indoor environments typically provide insufficient UV irradiance to fully activate TiO_2_-based coatings, suggesting that laboratory-reported antimicrobial performance may not directly translate to in-service conditions unless visible-light-active or doped systems are employed.^59^^,^^87^

### Role of interfacial phenomena in performance degradation

Across substrate classes, performance degradation mechanisms can be interpreted through interfacial phenomena relevant to organic coatings. On porous substrates, capillary depletion during application leads to non-uniform film thickness,^23^^,^^24^ while cyclic swelling and shrinkage induce interfacial stresses that promote cracking and delamination.^25^^,^^26^ Surface heterogeneity further exacerbates these effects by creating local variations in coating thickness and adhesion.^27^^,^^28^

On polymeric and composite substrates, additional degradation pathways arise from mismatch in thermal expansion coefficients and photooxidative degradation of the organic matrix.^77^^,^^81^ These mechanisms highlight the importance of matching coating mechanical properties (e.g., modulus, flexibility) to those of the substrate to maintain long-term adhesion and performance.

### Limitations and interpretation of findings

The findings of this study should be interpreted within the limitations of a structured scoping review following PRISMA-ScR guidance.^38^^,^^39^^,^^40^ The use of substrate class as a proxy for structural porosity enables cross-material comparison but does not constitute direct measurement of porosity. Consequently, the results represent associations between material type and performance outcomes rather than mechanistically confirmed relationships.

Similarly, the exploratory study-level regression approach provides directional insights but does not capture within-study variability or fully account for clustering effects. These limitations are addressed through conservative interpretation and sensitivity analyses, but precise effect sizes should be interpreted with caution.

The heterogeneity of testing protocols across studies—including variations in accelerated aging conditions, performance metrics, and reporting standards—further limits direct quantitative comparability. However, the consistency of observed trends across diverse studies supports the robustness of the substrate-related performance gradient.

### Implications for future research in coating science

The results identify several priorities for advancing coating science in this area. Direct experimental studies that systematically vary substrate porosity while holding coating chemistry and deposition conditions constant are required to establish direct experimental evidence linking porosity and performance.

Multilevel statistical models incorporating both study-level and observation-level variation would enable more precise quantification of chemistry effects. In addition, standardization of testing protocols—particularly for optical performance and accelerated aging—would improve comparability across studies.

Greater emphasis on simultaneous measurement of functional and optical properties from identical specimens is essential for evaluating real-world coating performance. Finally, long-term in-service studies are needed to bridge the gap between laboratory performance and operational durability, particularly in environments involving repeated cleaning, UV exposure, and mechanical wear.

### Practical implications for coating selection

From a practical perspective, the findings support a substrate-first approach to coating selection, consistent with materials selection frameworks.^18^ Substrate characteristics should be evaluated prior to formulation selection, with coating chemistry chosen to optimize performance within the constraints imposed by the substrate.

For non-porous substrates, coating selection can focus on balancing functional requirements (e.g., antimicrobial activity) with optical properties. For porous substrates, expectations for simultaneous high functional and optical performance should be moderated, and additional surface preparation or multi-layer strategies should be considered.

Overall, these results suggest that effective coating design requires integration of substrate properties, coating formulation, and application context within a unified framework grounded in interfacial science.

## Conclusion

This study identifies strong substrate-linked performance trends across heterogeneous nanocoating systems, with substrate class consistently emerging as an important predictor of dual functional-optical outcomes. Across 80 studies and 311 standardized observations, non-porous substrates consistently achieved substantially higher rates of simultaneous functional and optical success than highly porous materials, revealing a clear separation in performance between non-porous and highly porous materials. Exploratory study-level analysis supports this pattern, indicating significantly reduced odds of dual-axis success on highly porous substrates. These findings reframe nanocoating performance as an interfacial, substrate-dependent phenomenon rather than a formulation-driven outcome alone, supporting the importance of interfacial film formation processes in shaping coating performance.

Inclusion reliability was strengthened by post-hoc independent dual screening of all 105 full-text records (Cohen’s kappa = 0.947, almost perfect agreement), supporting confidence in the corpus as a rigorously verified foundation for these exploratory conclusions. Non-porous substrates (glass, glazed ceramics) achieved dual-axis success in 67–82% of studies in this corpus; highly porous substrates (wood, textiles) in only 11–22%. Exploratory study-level logistic regression (*N* = 80) with conservatively adjusted confidence intervals supports substrate class as showing the strongest observed association with dual-axis performance outcomes: highly porous substrates have 76% lower odds of dual-axis success than non-porous substrates (OR = 0.24; adjusted CI: 0.11–0.52).

Coating chemistry produces quantifiable trade-offs on non-porous substrates: TiO_2_ delivers superior antimicrobial efficacy (92% ≥ 4-log) at 12–15% greater gloss cost relative to SiO_2_; hybrid formulations show a promising descriptive pattern but rest on only five studies and cannot be treated as reliable guidance. Application context creates systematically divergent envelopes: healthcare achieves 87% ≥ 4-log antimicrobial efficacy at an 11.4 percentage point average gloss penalty; hospitality achieves 78% ≥ 92% gloss retention, but 74% of hospitality studies lack uncoated baselines, undermining attribution.

From a practical perspective, a substrate-first selection strategy is recommended. Substrate characteristics should be evaluated before coating chemistry is selected. For non-porous materials, coating choice should balance antimicrobial performance against optical preservation according to the intended application. For highly porous materials, expectations for simultaneous functional and optical performance should be moderated, and supplementary testing under deployment-representative conditions is recommended. No chemistry-specific recommendation is made for food preparation applications because the available evidence remains limited (*n* = 8 studies). These recommendations should be interpreted as practical guidance derived from the present synthesis rather than as prescriptive decision rules.

These conclusions are drawn from a scoping review whose inclusion decisions were verified by independent dual full-text screening (κ = 0.947), yet remain hypothesis-generating due to the proxy nature of porosity classification, study-level regression limitations, and absence of in-service longitudinal data.

Future work should combine controlled experimental studies with multilevel statistical approaches to better isolate the coupled contributions of substrate properties, coating chemistry, deposition conditions, and interfacial phenomena to coating performance.

## Acknowledgments

The authors acknowledge the financial support provided by the Deanship of Research and Graduate Studies at United Arab Emirates University (UAEU) for covering the open access publication fee associated with this article.

## Declaration of interests

The authors declare no competing interests.

## Supplemental information

Data S1. Study-level dataset and references for the 80 empirical studies included in the scoping review

## References

[1] Ulrich R.S., Zimring C., Zhu X., DuBose J., Seo H.B., Choi Y.S., Quan X., Joseph A. (2008). A review of the research literature on evidence-based healthcare design. HERD.

[2] Dancer S.J. (2014). Controlling hospital-acquired infection: Focus on the role of the environment and new technologies for decontamination. Clin. Microbiol. Rev..

[3] Emami M., Pazhouhanfar M., Stoltz J. (2024). Evaluating patients’ preferences for dental clinic waiting area design and the impact on perceived stress. Buildings.

[4] Hsu L.C., Fang J., Borca-Tasciuc D.A., Worobo R.W., Moraru C.I. (2013). Effect of micro- and nanoscale topography on the adhesion of bacterial cells to solid surfaces. Appl. Environ. Microbiol..

[5] Teughels W., Van Assche N., Sliepen I., Quirynen M. (2006). Effect of material characteristics and/or surface topography on biofilm development. Clin. Oral Implants Res..

[6] Lockyer T. (2003). Hotel cleanliness: How do guests view it?. Int. J. Hospit. Manag..

[7] Wicks Z.W., Jones F.N., Pappas S.P., Wicks D.A. (2007).

[8] Nguyen T., Collins B., Kaetzel L., Martin J., McKnight M. (1988).

[9] Kibert C.J. (2016).

[10] Faustini M., Nicole L., Ruiz-Hitzky E., Sanchez C. (2018). History of organic-inorganic hybrid materials. Adv. Funct. Mater..

[11] Novotny I., Flickyngerova S., Tvarozek V., Sutta P., Netrvalova M., Rossberg D., Schaaf P. (2014). Surface morphology and crystalline structure of sequentially sputtered ZnO nanocoatings. Appl. Surf. Sci..

[12] Law A.M., Jones L.O., Walls J.M. (2023). The performance and durability of anti-reflection coatings for solar module cover glass: A review. Sol. Energy.

[13] Ghamarpoor R., Jamshidi M., Joshaghani M. (2025). Hydrophobic silanes-modified nano-SiO_2_ reinforced polyurethane nanocoatings with superior scratch resistance, gloss retention, and metal adhesion. Sci. Rep..

[14] Burkovskaia K., Strankowski M., Szafran K. (2025). Synergistic effects of graphene and SiO2 nanoadditives on dirt pickup resistance, hydrophobicity, and mechanical properties of architectural coatings: A systematic review and meta-analysis. Coatings.

[15] Sanchez C., Julián B., Belleville P., Popall M. (2005). Applications of hybrid organic-inorganic nanocomposites. J. Mater. Chem..

[16] Makhlouf A.S.H., Tiginyanu I. (2011). Nanocoatings and Ultra-Thin Films: Technologies and Applications.

[17] Pavlič M., Petrič M., Žigon J. (2021). Interactions of coating and wood flooring surface system properties. Coatings.

[18] Ashby M.F. (2005).

[19] Wegst U.G.K., Ashby M.F. (2004). The mechanical efficiency of natural materials. Philos. Mag..

[20] Holmberg K., Matthews A. (2009). Techniques and Applications in Surface Engineering.

[21] Brinker C.J., Scherer G.W. (1990).

[22] Çöpoğlu N., Çiçek B. (2021). Abrasion resistant glass-ceramic coatings reinforced with WC-nanoparticles. Surf. Coat. Technol..

[23] Qiu Z., Tian G., Wang X. (2017). Investigation on stability and moisture absorption of superhydrophobic wood. Results Phys..

[24] Kanokwijitsilp T., Traiperm P., Osotchan T., Srikhirin T. (2016). Development of abrasion resistance SiO_2_ nanocomposite coating for teak wood. Prog. Org. Coat..

[25] Kamke F.A., Lee J.N. (2007). Adhesive penetration in wood—a review. Wood Fiber Sci..

[26] Wang F., Chen L., Zhang D. (2020). Superhydrophobic coatings on wood substrate for self-cleaning and EMI shielding. Appl. Surf. Sci..

[27] Pavlč M., Žigon J., Petrič M. (2021). The interactions between wood and coating constituents. Coatings.

[28] Prolongo S.G., Gude M.R., Ureña A. (2012). Nanoreinforced epoxy coatings for carbon fibre reinforced polymer composite structures. Prog. Org. Coating.

[29] Rowell R.M. (2012). Handbook of Wood Chemistry and Wood Composites.

[30] Hoadley R.B. (2000).

[31] Hossain M.M., Herrmann A.S., Hegemann D. (2006). Plasma hydrophilization effect on different textile structures. Plasma Process. Polym..

[32] Lettieri M., Masieri M., Frigione M. (2020). Durability to simulated bird guano of nano-filled oleo/hydrophobic coatings for the protection of stone materials. Prog. Org. Coat..

[33] Piispanen M., Määttä J., Areva S., Sjöberg A.-M., Hupa M., Hupa L. (2009). Chemical resistance and cleaning properties of coated glazed surfaces. J. Eur. Ceram. Soc..

[34] Aslanidou D., Karapanagiotis I., Lampakis D. (2018). Waterborne superhydrophobic and superoleophobic coatings for the protection of marble and sandstone. Materials.

[35] Yaghmour C.A., Marzouk M., Othman I. (2025). Advancing sustainable material selection in construction: A systematic review of MCDM applications. Decis. Making: Appl. Manage. Eng..

[36] Buyle M., Braet J., Audenaert A. (2013). Life cycle assessment in the construction sector: A review. Renew. Sustain. Energy Rev..

[37] Karana E., Pedgley O., Rognoli V. (2014). Materials Experience: Fundamentals of Materials and Design.

[38] Grant M.J., Booth A. (2009). A typology of reviews: An analysis of 14 review types and associated methodologies. Health Info. Libr. J..

[39] Tricco A.C., Lillie E., Zarin W., O’Brien K.K., Colquhoun H., Levac D., Moher D., Peters M.D.J., Horsley T., Weeks L. (2018). PRISMA Extension for Scoping Reviews (PRISMA-ScR): Checklist and explanation. Ann. Intern. Med..

[40] Barnett-Page E., Thomas J. (2009). Methods for the synthesis of qualitative research: A critical review. BMC Med. Res. Methodol..

[41] Page M.J., McKenzie J.E., Bossuyt P.M., Boutron I., Hoffmann T.C., Mulrow C.D., Shamseer L., Tetzlaff J.M., Akl E.A., Brennan S.E. (2021). The PRISMA 2020 statement: An updated guideline for reporting systematic reviews. BMJ.

[42] ISO 19403-2:2020. Paints and Varnishes — Wettability — Part 2: Determination of the Surface Free Energy of Solid Surfaces by Measuring the Contact Angle. International Organization for Standardization.

[43] ASTM D4060-19. Standard Test Method for Abrasion Resistance of Organic Coatings by the Taber Abraser. ASTM International.

[44] ASTM E2180-18. Standard Test Method for Determining the Activity of Incorporated Antimicrobial Agent(s) in Polymeric or Hydrophobic Materials. ASTM International.

[45] ISO 11664-4:2008. Colorimetry — Part 4: CIE 1976 L∗a∗b∗ Colour Space. International Organization for Standardization.

[46] ASTM D2244-16. Standard Practice for Calculation of Color Tolerances and Color Differences from Instrumentally Measured Color Coordinates. ASTM International.

[47] ISO 2813:2014. Paints and Varnishes — Determination of Gloss Value at 20°, 60° and 85°. International Organization for Standardization.

[48] Hunt R.W.G., Pointer M.R. (2011).

[49] Joanna Briggs Institute (2020). JBI Manual for Evidence Synthesis.

[50] Popay J., Roberts H., Sowden A., Petticrew M., Arai L., Rodgers M., Duffy S. (2006).

[51] Cai L., Ni C., Chen H. (2022). UV-resistant visually transparent nanocoatings on wood substrate. Prog. Org. Coating.

[52] Gholamiyan S., Hamzeh Y., Ashori A. (2024). Multifunctional PVB nanocomposite wood coating by cellulose nanocrystal/ZnO nanofiller. Int. J. Biol. Macromol..

[53] Vélez C., Palma A., Aguilera A., Ananias R.A. (2022). Nanocomposite additive of SiO_2_/TiO_2_/nanocellulose on waterborne coating formulations. Case Stud. Constr. Mater..

[54] Mazur M., Wojcieszak D., Kaczmarek D., Domaradzki J., Song S., Gibson D., Placido F., Mazur P., Kalisz M., Poniedzialek A. (2016). Functional photocatalytically active and scratch resistant antireflective coating based on TiO_2_ and SiO_2_. Appl. Surf. Sci..

[55] Ghorban F., Eshaghi A. (2017). Environmental effects on transparency and hydrophilicity of silica nano-porous thin film coated glass substrates. Opt. Quantum Electron..

[56] Miao L., Su L., Tanemura S., Fisher C.A.J., Zhao L., Liang Q., Xu G. (2013). Cost-effective nanoporous SiO_2_–TiO_2_ coatings on glass substrates. Appl. Energy.

[57] Ke C., Zhang C., Jiang Y. (2023). Robust transparent superhydrophobic coatings on glass substrates. Ceram. Int..

[58] Ke C., Zhang C., Jiang Y., Wang S. (2025). Transparent superhydrophobic and thermal insulating dual-functional coatings. Int. J. Appl. Ceram. Technol..

[59] Garlisi C., Trepci E., Li X., Al Sakkaf R., Al-Ali K., Nogueira R.P., Palmisano G. (2020). Multilayer thin film structures for multifunctional glass. Appl. Energy.

[60] Fardo F.M., Ribeiro R.S., Strauss J.A. (2022). Double layer SiO_2_–TiO_2_ sol–gel thin films on glass. Results Phys..

[61] Franceschin G., Zendri E., Casadio F., Bultrini G. (2025). Enhancing environmental stability and transparency of glass coatings using silica nanoparticles. Adv. Mater. Interfac..

[62] Li Z., He H., Wang X., Shou C., Huang M., Jin S., Du X. (2023). Robust SiO_2_@TiO_2_ nanocoatings with antireflection and photocatalytic self-cleaning properties. Colloids Surf., A.

[63] Zhan Y., Zhou H., Guo F., Li S., Ma W., Tian Y., Jia S. (2019). Antiglare and antireflective coating of layer-by-layer SiO_2_ and TiZrO_2_ on glass. Appl. Surf. Sci..

[64] Yu H., Qin Y., Chen B., Song F., Deng S., Wen L., Pan J., Hu D. (2025). Long-lasting antibacterial fabrics with hydrophobic, self-cleaning, and blood stain-resistant properties fabricated by ZnO@ OTS coating. Int. J. Biol. Macromol..

[65] Perelshtein I., Applerot G., Perkas N., Wehrschetz-Sigl E., Hasmann A., Guebitz G.M., Gedanken A. (2009). Antibacterial properties of ZnO nanocrystalline deposited on fabrics. ACS Appl. Mater. Interfaces.

[66] Perelshtein I., Ruderman Y., Perkas N., Traeger K., Tzanov T., Beddow J., Joyce E., Mason T.J., Blanes M., Mollá K., Gedanken A. (2012). Enzymatic pre-treatment as a means of enhancing the antibacterial activity and stability of ZnO nanoparticles sonochemically coated on cotton fabrics. J. Mater. Chem..

[67] Salat M., Petkova P., Hoyo J., Perelshtein I., Gedanken A., Tzanov T. (2018). Durable antimicrobial cotton textiles coated sonochemically with ZnO nanoparticles. Carbohydr. Polym..

[68] Prasad V., Arputharaj A., Bharimalla A.K., Patil P.G., Vigneshwaran N. (2016). Durable multifunctional finishing of cotton fabrics by in situ synthesis of nano-ZnO. Appl. Surf. Sci..

[69] Salama K.F., AlJindan R., Alfadhel A., Akhtar S., Al-Suhaimi E.A. (2024). Enhanced antimicrobial performance of textiles coated with TiO_2_ nanoparticles. Textil. Res. J..

[70] Islam M.S., Khan M.A., Akter M. (2022). Fabrication and utilization of ZnO nanoparticles for stain release, bacterial resistance, and UV protection on cotton fabric. Textil. Res. J..

[71] Vaiano V., Sarno G., Sannino D., Ciambelli P. (2015). Photocatalytic properties of TiO_2_-functionalized tiles. Res. Chem. Intermed..

[72] Sciancalepore C., Bondioli F. (2015). Durability of SiO_2_–TiO_2_ photocatalytic coatings on ceramic tiles. Int. J. Appl. Ceram. Technol..

[73] Murugan K., Subasri R., Rao T.N., Gandhi A.S., Murty B.S. (2013). Synthesis, characterization and demonstration of self-cleaning TiO_2_ coatings on glass and glazed ceramic tiles. Prog. Org. Coat..

[74] Bianchi C.L., Gatto S., Pirola C., Naldoni A., Di Michele A., Cerrato G., Crocellà V., Capucci V. (2014). Photocatalytic degradation of acetone, acetaldehyde and toluene in gas-phase: Comparison between nano and micro-sized TiO_2_. Appl. Catal. B Environ..

[75] Vaiano V., Sarno G., Ciambelli P., Sannino D. (2014). Functionalization of ceramic tiles with N-doped TiO_2_ for photocatalytic function. J. Adv. Oxid. Technol..

[76] De Niederhäusern S., Bondi M., Bondioli F. (2013). Antibacterial ceramic surfaces: Overview of test methods. Int. J. Appl. Ceram. Technol..

[77] Zhu M., Qian H., Zhang L., Li L., Wang C., Kang J., Lu C., Gao Q. (2025). Synergistic enhancement of anti-scaling performance in epoxy coatings through surface modification of h-BN and nano-SiO_2_. Colloids Surf. A Physicochem. Eng. Asp..

[78] Malaki M., Hashemzadeh Y., Karevan M. (2016). Effect of nano-silica on the mechanical properties of acrylic polyurethane coatings. Prog. Org. Coat..

[79] Jalili M.M., Moradian S., Dastmalchian H., Karbasi A. (2007). Investigating the variations in properties of 2-pack polyurethane clear coat through separate incorporation of hydrophilic and hydrophobic nano-silica. Prog. Org. Coat..

[80] Ramezanzadeh B., Attar M.M., Farzam M. (2011). A study on the anticorrosion performance of the epoxy–polyamide nanocomposites containing ZnO nanoparticles. Prog. Org. Coat..

[81] Amerio E., Fabbri P., Malucelli G., Messori M., Sangermano M., Taurino R. (2008). Scratch resistance of nano-silica reinforced acrylic coatings. Prog. Org. Coat..

[82] Zhao Y., Xu Z., Wang X., Lin T. (2013). Photoreactive isobutyl POSS reinforced polyurethane nanocomposite coatings. Prog. Org. Coating.

[83] Rashvand M., Ranjbar Z., Rastegar S. (2011). Nano zinc oxide as a UV-stabilizer for aromatic polyurethane coatings. Prog. Org. Coat..

[84] Asmatulu R., Mahmud G.A., Hille C., Misak H.E. (2011). Effects of UV degradation on surface hydrophobicity, crack, and thickness of MWCNT-based nanocomposite coatings. Prog. Org. Coat..

[85] Liu H., Shang J., Chen X., Kamke F.A., Guo K. (2019). Effect of nanosilica content in coextruded wood-plastic composites on ultraviolet aging resistance. Polym. Adv. Technol..

[86] Allen N.S., Edge M., Ortega A., Sandoval G., Liauw C.M., Verran J., Stratton J., McIntyre R.B. (2004). Degradation and stabilisation of polymers and coatings: Nano versus pigmentary titania particles. Polym. Degrad. Stab..

[87] Kapridaki C., Pinho L., Mosquera M.J., Maravelaki-Kalaitzaki P. (2014). Producing photoactive, transparent and hydrophobic SiO_2_–crystalline TiO_2_ nanocomposites. Appl. Catal., B.

[88] Heinonen S., Huttunen-Saarivirta E., Nikkanen J.-P., Raulio M., Välimäki M., Laakso J., Storgårds E., Levänen E. (2014). Antibacterial properties and chemical stability of superhydrophobic silver-containing surface produced by sol–gel route. Colloids Surf. A Physicochem. Eng. Asp..

